# Once upon a time: the glucagon stimulation test in diagnosing adult GH deficiency

**DOI:** 10.1007/s40618-024-02322-5

**Published:** 2024-03-10

**Authors:** D. Cuboni, M. Caputo, E. Ghigo, G. Aimaretti, V. Gasco

**Affiliations:** 1 Division of Endocrinology, Diabetes and Metabolism, Department of Medical Science, ASOU “Città della Salute e Della Scienza” di Torino, University of Turin, C.So Dogliotti 14, 10126 Turin, Italy; 2 Department of Health Sciences, Università del Piemonte Orientale, Novara, Italy; 3 Endocrinology, Department of Translational Medicine, Università del Piemonte Orientale, Novara, Italy

**Keywords:** Glucagon stimulation test, Adult GH deficiency, Diagnosis, Accuracy, Sensibility, Specificity

## Abstract

**Purpose:**

The clinical features of adult GH deficiency (GHD) are nonspecific, and its diagnosis is established through GH stimulation testing, which is often complex, expensive, time-consuming and may be associated with adverse side effects. Moreover, diagnosing adult GHD can be challenging due to the influence of age, gender, and body mass index on GH peak at each test. The insulin tolerance test (ITT), GHRH + arginine test, glucagon stimulation test (GST), and, more recently, testing with macimorelin are all recognized as useful in diagnosing adult GHD. To date GST is still little used, but due to the unavailability of the GHRH all over the world and the high cost of macimorelin, in the next future it will probably become the most widely used test when ITT is contraindicated. The aim of the present review is to describe the current knowledge on GST.

**Methods:**

Narrative review.

**Results:**

In the last years several studies have suggested some changes in the original GST protocol and have questioned its diagnostic accuracy when the classic GH cut-point of 3 μg/L is used, suggesting to use a lower GH cut-point to improve its sensitivity and specificity in overweight/obese patients and in those with lower pretest GHD probability.

**Conclusion:**

This document provides an update on the utility of GST, summarizes how to perform the test, shows which cut-points should be used in interpreting the results, and discusses its drawbacks and caveats referring to the most recent studies.

## Introduction

**Fig. 1:**
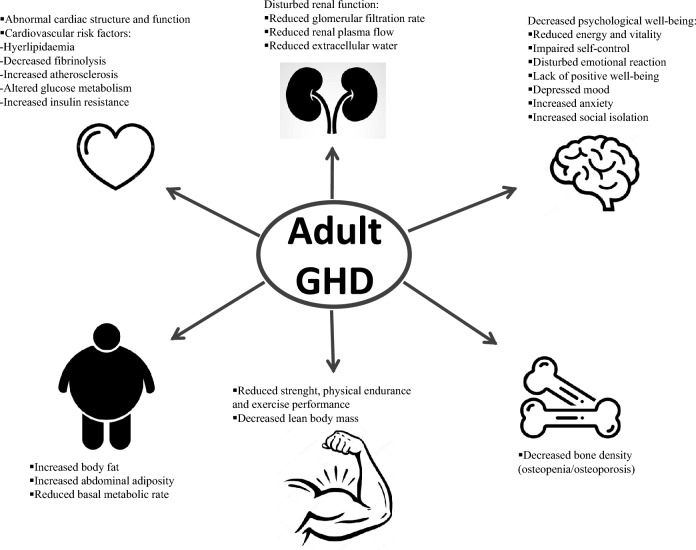
Clinical picture of adult growth hormone deficiency (GHD)

Adult growth hormone deficiency (GHD) is a clinical condition characterized by a decreased growth hormone (GH) secretion from the anterior pituitary. This condition is characterized by metabolic impairment, i.e., alterations in body composition, insulin resistance and dyslipidemia, endothelial dysfunction with premature atherosclerosis, and decreased muscle strength and exercise capacity, which together result in diminished quality of life [1] (Fig. 1). Moreover, GHD seems to contribute to the cardiovascular morbidity and mortality that is increased in patients affected by hypopituitarism compared to the general population [2]. In the instance of suspicion of adult GHD, performing the diagnosis is essential if the intention to start recombinant human GH (rhGH) replacement therapy is established.

The diagnosis of GHD requires GH-stimulation tests, except for patients affected by: i) organic hypothalamic–pituitary disease with ≥ 3 pituitary hormone deficiencies and serum IGF-I levels < − 2.0 SDS; ii) genetic defects of the hypothalamic–pituitary axes; iii) hypothalamic–pituitary structural brain defects, in which additional tests are not necessary [3].

The insulin tolerance test (ITT) has been historically considered the gold standard for diagnosis; nevertheless, the need to achieve hypoglycemia with the potential life-threatening side effects and the requirement of medical supervision make this test not feasible in frail populations (i.e., elderly, and patients with history of or at risk of seizures and/or stroke and cardiovascular diseases). If ITT is contraindicated, a reliable alternative is GH-releasing hormone (GHRH) + arginine (ARG) test, using BMI-related GH cutoffs for the interpretation of results [4, 5]. In fact, overweight and obesity are known to be conditions of relative GHD, related to reduced GH half-life, fewer GH pulses, and longer intervals between GH pulses [6]. However, since the production of recombinant GHRH has been interrupted in the United States (US) in 2008 (Serono, 2008, http://www.fda.gov/cder/drug/shortages/GerefDiagnosticDiscontinuationLetter.pdf), and in Europe in 2023, a significant gap had remained on the diagnosis of GHD when ITT is contraindicated.

The other tests suggested by guidelines are the glucagon stimulation test (GST) and the macimorelin test [3]. Macimorelin is a synthetic GH secretagog (GHS) [7] that has the advantage to be orally administered, with few side effects [8]. Unfortunately, very high cost and the potential drug-to-drug interactions limit the use of the test. Moreover, the announcement in August 2022 that macimorelin should be temporarily discontinued in the commercial market of US makes this test less used in clinical practice (https://www.globenewswire.com/en/news-release/2022/08/29/2505902/0/en/Aeterna-Zentaris-Set-to-Regain-Full-Rights-to-Macrilen-Macimorelin-in-U-S-and-Canada-from-Novo-Nordisk.html). Thus, the GST has increased as an alternative test to asses GH reserve in adults [9]. The use of this test has increased since 2009, reflecting guidelines and the lack of GHRH [10], even though the use is still limited [11, 12]. Thus, the aim of the present review is to describe the current knowledge on GST (practical information on test execution methods, classical, and BMI-related cutoffs, potential side effects).

## Update on GST in diagnosing adult GHD

### Physiology of GST

The physiological mechanism underlying glucagon's ability to stimulate the GH (and ACTH) secretion has yet to be conclusively elucidated. Since the 1970s, when the polypeptide's stimulatory properties were first demonstrated, several hypotheses have been proposed.

The initial observed phenomenon was an increase in blood glucose (BG) levels within the first hour after subcutaneous (sc) or intramuscular (im) administration of glucagon, followed by a subsequent rapid decrease due to insulin stimulation [13]. Initially, it was postulated that these glycemic fluctuations were the trigger for GH release [14, 15]. However, the effectiveness of GH stimulation in diabetic subjects, even in the absence of substantial glycemic variations, has underscored the possibility of alternative mechanisms [16, 17]. Moreover, the glycemic threshold identified for eliciting the counterregulatory GH response is 40 mg/dL. Recent data also demonstrate that although significant glycemic variations occur after glucagon administration, the nadir falls well above this threshold [18, 19]. Although the data may be controversial, it is evident that glycemic levels, circulating free fatty acids (FFA), and insulin levels continue to hold significance in the identification of GHD patients. Andersen et al. demonstrate that the response to the GST is lower in healthy obese subjects with chronically elevated FFA and hyperinsulinemia. In these individuals, the administration of nicotinic acid, a potent inhibitor of FFA release, or the use of pioglitazone, was able to enhance the performance of the test [20].

Following glucagon administration, symptoms like nausea, restlessness, and vomiting have been observed, leading to the hypothesis of induced stress as a potential stimulus [21]. However, stress-related symptoms are more pronounced with intravenous (iv) administration of glucagon, which has been shown to be less effective in eliciting a secretagog action on the somatotropic axis [21].

An intriguing hypothesis assigns a central role to the release of catecholamines, particularly noradrenaline. After sc administration of glucagon, a biphasic trend in blood concentrations of noradrenaline has been observed. An initial peak of noradrenaline occurs after 30–60 min, in conjunction with the peak of BG and insulin, followed by a second peak at 120–180 min, coinciding with the rise in GH and cortisol levels, and the appearance of side effects experienced by subjects. However, the same authors did not find a correlation between the peak levels of hormones and the reported severity of symptoms [22]. Additional support for the role of noradrenaline is provided by the observation that simultaneous administration of beta blockers enhances the response to glucagon, indicating a central involvement in the stimulation of alpha-adrenergic receptors [23, 24].

The observation of a lack of GH (and ACTH) stimulation following iv administration [25] has prompted the hypothesis that glucagon may not inherently act as a secretagog. Instead, the stimulatory role might be attributed to a fragment of glucagon generated through peptide proteolysis after im administration.

GHSs encompass both peptides and non-peptides, including substances such as ghrelin, macimorelin, and hexarelin, all of which exert a potent stimulatory effect. Arvat et al. have demonstrated that the im administration of glucagon and hexarelin synergistically stimulates the somatotropic axis, while exhibiting a less than additive effect on corticotropin secretion. This finding suggests that the action of the peptidic fragment of glucagon, acting as a secretagogue, may operate via a similar mechanism to hexarelin concerning the corticotropin axis [26]. However, the mechanisms of glucagon-induced GH stimulation remains unclear, and one hypothesis is that glucagon decreases ghrelin-independent effects of glucose or insulin variations [27].

Furthermore, recent research has explored the interaction between glucagon and a potent endocrine regulator known as fibroblast growth factor-21 (FGF-21). This protein is primarily produced in the liver, but also in the pancreas, adipose tissue, and skeletal muscle, exerting effects on carbohydrate and lipid metabolism akin to those of glucagon [28]. Glucagon has been shown to increase hepatic secretion of FGF-21 [29], and some of its metabolic effects are thought to occur, at least partially, through the FGF-21-dependent pathway [30]. However, Akkar et al. did not find a correlation between the rise in FGF-21 levels after glucagon administration and the increase in GH and cortisol, suggesting that glucagon's effects on the somatotropic and hypothalamic–pituitary–adrenal axes occur independently of FGF-21 [28].

Despite the numerous hypotheses put forth, further studies are required for a more comprehensive understanding of the physiological mechanism underlying glucagon's stimulation of the somatotropic axis.

### GST: protocol and considerations

Glucagon injection as a GH provocative test has been studied extensively since its initial report in 1969 [13, 15]. The test has gained widespread acceptance in the United Kingdom (UK), where it is the second most popular GH provocative test after the ITT, but until recently it had been used relatively infrequently in the US. In recent years, the use of GST has also grown in the US due to the withdrawal of GHRH from the market, necessitating the adoption of GST as an alternative. The American Association of Clinical Endocrinology (AACE) and the American College of Endocrinology (ACE) have proposed a standardized protocol.

The test should be conducted between 8 and 9 a.m., and patients should fast for at least 8–10 h beforehand. Upon arrival, weight and fasting BG levels should be assessed. Specifically, fasting BG levels exceeding 180 mg/dL constitute a contraindication to initiating the test. Patients should be informed of potential side effects, and the administration of anti-emetics may be necessary.

The protocol entails administering 1 mg of glucagon im (1.5 mg if the patient weights > 90 kg), with serial venous blood samples taken through a cannula every 30 min for 4 h. In particular, both serum GH levels and venous BG should be measured at 0, 30, 60, 90, 120, 150, 180, 210, and 240 min. The patient should remain in a supine position during the test. Given the potential for delayed hypoglycemia, patients should be advised to consume small, frequent meals after completing the test [31]. BG usually rises to peak around 90 min and then gradually declines. To date, glycemic variations recorded during GST are not used for the interpretation of the results, but some works [32, 33] have suggested the need to review this position.

Regarding the dosage of glucagon to administer for maximizing the stimulatory effect, Yuen et al. compared the weight-based regimen (WB, 0.03 mg/kg) with the fixed-dose regimen (FD, 1–1.5 mg). This study showed that both the WB and FD regimens were capable of inducing peaks in GH in adults. However, in the WB regimen, a later peak was observed alongside more pronounced side effects. As age, BMI, and BG levels can influence the test, the authors suggested using the FD in young normoglycemic subjects, while the WB may be more beneficial for older patients with glucose intolerance [33].

Currently, in the absence of solid data, and considering the challenges in interpreting the test, it is recommended to administer the test at FD of 1 mg of glucagon for subjects weighting less than 90 kg, and 1.5 mg for those weighting more.

Over the years, there have been ongoing endeavors to refine the test protocol, including exploring alternative administration routes.

Ghigo et al. were at the forefront of demonstrating the inefficacy of somatotropic axis stimulation via iv glucagon administration [25]. Moreover, a recent attempt to alter the mode of delivery involved investigating intranasal administration. However, even with this adjustment, it did not yield a significant response in either the somatotropic or corticotropic axis [34].

The GST was initially described as a 4 h test, but some efforts have been made to make the test more practical, focusing mainly on reducing the time required to perform it.

Some authors have suggested shortening the test to 3 h and reducing the number of sample points to just three or five (0, 90, 120, 150, and 180 min), given that the majority of GH peaks occur between 120 and 180 min [19, 35]. Orme et al. compared the standard protocol with a shorter version, which involved assessments at three time points (0, 150, and 180 min) following im glucagon administration. They noted that in 75% of the cases with conflicting results, a peak was detected at 210 min. Consequently, these authors proposed extending the test to at least 210 min [35]. An audit involving 500 patients with pituitary disease suggested shortening the test by omitting the 240 min sample. The majority of patients exhibited a peak between 120 and 180 min (85%), whereas only five subjects demonstrated it at 240 min [19]. The timing of the GH peak to GST was also examined in 105 subjects within 4 h by Ditchel [36]. Most GH peaks occurred within the third hour of the GST. However, 14% of all subjects, including 13% of all controls, 18% of patients with partial pituitary deficiencies, and 10% of patients with total pituitary deficiencies had a GH peak in the 4^th^ hour of the test. Detailed analysis showed that no subjects would be reclassified as failing the GST if GH levels from the 1^st^ hour of testing were excluded. Additionally, no subjects would be reclassified if only the last half-hour of testing were omitted. However, three subjects who initially passed the GST would be reclassified as failing if the last full hour of testing (210 and 240 min) were omitted [36]. Similarly, Diri et al. [37] showed how the GH peak to GST occurs at 210 and 240 min in 31.5% of the subjects studied.

Considering the short regimen from another point of view, Yuen et al. highlight the risk, with a short regimen, of the lack of evidence for delayed hypoglycemia, despite levels below the risk threshold being rare [38].

Even if the ideal timing for sample collection, ensuring the best balance between the test accuracy and feasibility, remains unclear, overall, to avoid overdiagnosis of GHD, these data would suggest that it is prudent to extend the evaluation to 240 min, while it seems reasonable to omit the sampling at 0, 30, and 60 min without compromising the diagnostic accuracy of the test.

To enhance the diagnostic test's power, several protocols combining glucagon with other agents have been proposed. The initial proposal came from the group of Mitchel et al. in 1971, who observed an amplified response to the test by co-administering 40 mg of propranolol [24]. More recent data pertain to the combination of GST with pegvisomant (PegV), a GH-receptor antagonist capable of increasing circulating GH levels. The authors demonstrated that a single dose of PegV, when priming the GST, rapidly induces an elevation in basal GH and GHBP levels, reducing IGF-I levels. Although an actual enhancement in test accuracy was not demonstrated, a strong positive correlation was noted between serum PegV levels and basal and peak GH levels [31].

However, none of these schemes are currently utilized in clinical practice.

## Old versus new BMI-related cutoffs

The diagnostic accuracy of GST in identifying GHD was assessed in three studies [39–41]. Gomez et al. [40] evaluated 119 adult subjects (73 GHD diagnosed by ITT and 46 healthy controls), and demonstrated that GST (glucagon 1 mg im or 1.5 mg if body weight was > 90 kg) had 100% sensitivity and 100% specificity using a peak of 3 µg/L. Moreover, the authors underlined a negative correlation between age and BMI in healthy controls. Conceicao et al. [41] performed GST (1 mg im) in 58 adult subjects (33 GHD diagnosed by ITT and 25 healthy controls matched for age and sex) showing that GH peak of 3 µg/L was associated with 97% sensitivity and 88% specificity. Notably, unlike the previous study, GH peak levels were not influenced by age and gender. Finally, in the study by Berg et al. [39], 49 post-surgery adult GHD patients diagnosed by ITT underwent GST (glucagon 1 mg im or 1.5 mg if body weight was > 90 kg), and 2.5 µg/L was the GH peak cutoff showing 95% sensitivity and 79% specificity, without correlation with BMI or age.

Subsequent research hypothesized that the GH peak of 3 µg/L could lead to an overdiagnosis of GHD in overweight and obese patients. In particular, a retrospective study [36] evaluated 108 overweight and obese adult male subjects, divided into three groups: 20 patients affected by total pituitary insufficiency (3–4 hormonal deficits), 41 by partial pituitary deficits (1–2 hormonal deficits), and 47 healthy controls. The results demonstrated that a GH peak equal to 0.94 µg/L had the highest sensitivity (90%) and specificity (94%) and that BMI and visceral adipose tissue were negatively correlated to GH peak in controls.

Another study in a large Turkish population [37] (216 patients affected by pituitary disorders and 26 healthy controls) investigated the optimal GH cutoff to GST (1.0 mg im and 1.5 mg if body weight was > 90 kg) compared to ITT. The authors considered two GST cutoffs as diagnostic of GHD (3.0 and 1.07 µg/L). The results showed that all patients with pituitary disease showing sufficient GH response to ITT had an adequate response to GST with either 3.0 or 1.07 µg/L cutoff, as well as patients with an inadequate GH response. On the contrary, 12 out of 26 (46.2%) healthy subjects failed the GST using a GH cut point of 3.0 µg/L, but none did when the cut point was lowered to 1.07 µg/L. Thus, in ROC curve analysis 1.07 µg/L GH peak cutoff had 100% specificity and 100% sensitivity, while GST with 3.0 µg/L cutoff had 100% sensitivity but 84% specificity. In addition, excluding from the analysis patients with more than three pituitary deficits, BMI negatively correlated with GH peak concentrations on ITT and GST.

Wilson et al. [32] evaluated GST (1.0 mg im and 1.5 mg if body weight was > 90 kg) in 42 patients characterized by a high pre-test probability of GHD; of them, 29% was overweight and 62% was obese, without differences in anterior pituitary hormone deficits in obese than non-obese patients. Obese patients (*N* = 26) had a lower GH response at GST than non-obese, demonstrated by a lower GH area under the curve (AUC). In particular, obese women (*N* = 19), had a lower GH peak than non-obese ones (2.02 ± 2.80 vs. 5.39 ± 4.68 µg/L). Moreover, excluding from the analysis ten patients with severe GHD (GH peak ≤ 0.1 μg/L), body weight negatively correlated with GH AUC and peak GH response.

A more recent research evaluated the best GH peak cutoff to reduce overdiagnosis of GHD in overweight and obese patients during GST at FD (1 mg or 1.5 mg if body weight was > 90 kg) or WB dose (0.03 mg/kg) using ITT as gold standard [42]; the study included 28 adult patients with hypothalamic–pituitary disease and 14 healthy controls matched for age, sex, BMI and estrogen status, and proposed a GH peak cutoff of 1.0 µg/L for FD (92% sensitivity and 100% specificity) and 2.0 µg/L for WB dose (96% sensitivity and 100% specificity). Notably, a negative correlation of GH peak and age in FD GST, but not in WB dose GST was found.

In light of these findings, the AACE guidelines recommend utilizing BMI-appropriate peak GH cut points for the GST to diagnose adult GHD to reduce the possibility of GHD overdiagnosis, especially in overweight and obese subjects (Table 1). For normal weight (BMI < 25 kg/m^2^) and overweight (BMI 25–30 kg/m^2^) patients with a high pretest probability, they recommend using the GH cut point of 3 µg/L, whereas for obese (BMI > 30 kg/m^2^) and overweight (BMI 25–30 kg/m^2^) patients with a low pretest probability, they recommend using a lower GH cut point of 1 µg/L [3].

**Table 1:** GH cutoffs to glucagon stimulation test (GST) in transition and adult patients with clinical suspicion of growth hormone deficiency (GHD) as per AACE guidelines

Subjects	GH cutoff (µg/L)
Normal weight (BMI < 25 kg/m^2^) transition and adult patients	≤ 3
Overweight (BMI 25–30 kg/m^2^) transition and adult patients with a high pre-test GHD probability	≤ 3
Overweight (BMI 25–30 kg/m^2^) transition and adult patients with a low pre-test GHD probability	≤ 1
Obese (BMI > 30 kg/m^2^) transition and adult patients	≤ 1

## GST drawbacks

The GST is recognized as a viable alternative to the ITT, particularly due to its relative safety profile with minimal contraindications. The AACE identifies only two established contraindications [31]:

- Malnutrition or extended fasting for over 48 h, owing to the metabolic impact of the GST.
- Fasting BG levels over 180 mg/dL at the test's commencement, both for the risk of initial deterioration and for doubts regarding the test's efficacy in cases of marked hyperglycemia.

Given the glycemic fluctuations induced by glucagon and the subsequent rebound insulin, diligent glucose level monitoring throughout the test is imperative to detect any early hyperglycemia or late hypoglycemia [31].

However, also a history of pheochromocytoma is an absolute contraindication. This is due to concerns of potential blood pressure instability and the risk of a catecholaminergic crisis, triggered by the noradrenaline release induced by glucagon.

Insulinoma has also been reported as a contraindication to GST in some [40, 43–45], but not all papers [3, 9, 31, 38, 46].

The primary reported side effects during the test encompass nausea, vomiting, and headache, with frequencies ranging from < 10 [39] to 34% [19], predominantly occurring between 60 and 120 min. These side effects typically resolve by the 240 min mark [33].

However, special attention must be given to elderly subjects, a demographic in which the test has received less comprehensive study [47]. Valuable safety insights have emerged from a study conducted by Winter-Tavares et al. in 2015. Among a sample of 42 healthy subjects aged between 67 and 87 years, 9 subjects experienced side effects during the test. In addition to the known effects, in four cases marked hypotension occurred, alongside disabling dizziness in one subject and tonic–clonic seizures in another [48]. Also in Hamrahian's study, a case of epileptic seizure was reported in a subject with no previous history of seizure disorder [42].

A prior study by Rao and Spathis also detailed transient bradycardia and mild hypotension, associated with severe retching and vomiting, pallor, and sweating in 10% of the studied population [43]. The side effect was attributed to the pronounced release of noradrenaline. Notably, given noradrenaline's substantial affinity for ß1 receptors with a positive inotropic and chronotropic effect, intense vagal stimulation could potentially occur to counterbalance, a phenomenon more pronounced in the elderly due to their heightened basal vagal tone [48].

Taking these data into consideration, it may be useful to assess heart rate and blood pressure during the test.

In relation to recent research from our group with the objective to evaluate the response following intranasal glucagon stimulation [34], concerning data have come to light. We noted a significant reduction in potassium levels, reaching a nadir at 45 min, coinciding with the insulin peak. Specifically, six out of ten subjects developed hypokalemia, with a lowest value of 2.9 mmol/L [34]. While electrolyte monitoring is not mandated by guidelines, the measurement of sodium and potassium levels could be considered to assess for fluctuations following im glucagon administration.

In conclusion, GST is generally considered low-risk at present, but future large-scale studies, especially in light of recent evidence, will help elucidate any aspects not previously considered.

## Caveats in performing and interpreting the GST

Considering the high cost of the replacement treatment with rhGH and its potential long-term risks, it is essential to establish the correct diagnosis so that appropriate GH replacement is offered only to adults who are truly GHD. The diagnosis of adult GHD is challenging for the clinician because of the lack of a single biological end point. Adult GHD diagnosis depends on the demonstration of a reduced peak serum GH level in response to one or more GH stimulation tests [3, 49–51]. Currently, there is no ideal stimulation test and the decision to consider performing a GH stimulation test to diagnose adult GHD must take into account the validity of the chosen test, its GH cutoffs, the availability of local resources and of stimulant agent, and the clinician expertise.

In recent years, also following the withdrawal of GHRH from the market, GST is increasingly used as the alternative test to ITT. This is due to the relatively low cost of GST and its safety.

However, it is necessary to consider some caveats in the use and interpretation of GST as it is used today.

Initially, the diagnostic GST cutoffs were identified by looking for the minimal response observed in a group of normal subjects [52–55]; subsequently new studies have revisited the diagnostic criteria comparing the response to GST with that of the test considered the gold standard (mostly ITT) [37, 39–42], often comparing patients with normal subjects [37, 40–42]. Anyway, all these approaches are exposed to possible bias. In particular, the studies that compare the response of a group of patients already diagnosed for the target condition with the response observed in healthy volunteers, even if matched by sex, age and BMI, are at risk of overestimating the diagnostic accuracy of the cutoffs thus identified and, consequently, the results obtained cannot be applied to the clinical setting. On the other hand, studies that establish the diagnostic accuracy of a particular test, comparing it with another diagnostic test, are at risk of overestimating or underestimating the diagnosis similarly to what the gold standard itself does.

Furthermore, it should be considered that even the most recent studies that have re-evaluated the GST cutoffs as a function of BMI [36, 37, 42], have been conducted on small groups of subjects with the consequent need for further validation of the results obtained on a larger scale.

Another factor to keep in mind when interpreting the results of the several studies performed over the years is that the GH cut points suggested in any study depend on the assay method used. Variability between assay results may exceed 100% in many cases, limiting the applicability of assay-specific cut points for routine clinical practice [56]. Among the reasons for the variation in the GH assay results include the heterogeneity of the analyte itself, the availability of different preparations for calibration, and the potential interference from other components such as GHBP [57]. However, this is true for every assay and every stimulation test. Accordingly, both test- and assay-specific cutoffs are of special importance to avoid misinterpretation, so caution is required before adopting or extrapolating cutoff values from other laboratories [56, 58, 59].

Reports on whether there is a correlation between peak GH with other factors in addition to BMI, as age, and gender during GST are mixed (Table 2). Consequently, the use of the same diagnostic thresholds to GST across all adult age groups is currently questionable. It is well known that GH secretion decreases physiologically with aging [60]. To date it is not yet clear if the GH response to the GST is age dependent [61]: the age of the patient did not affect the GH peak achieved during GST in the study by Toogood [11] and by Wilson [32]; however, not all reports are in agreement with such findings showing a negative correlation between age and peak GH levels during GST [33, 37, 40, 42]. Anyway, to date, the diagnostic accuracy of GST has not been sufficiently investigated in the extreme ranges of what can be considered adulthood: it is therefore of fundamental importance to evaluate the diagnostic performance of GST in the transition phase by looking for the most appropriate cutoffs in this particular life’s phase; moreover, it should be considered that none of the studies carried out so far on GST have been performed in elderly patients (age > 70 years) and this makes it difficult to extrapolate the results obtained in a young population to an elderly one, in whom underlying co-morbidities may be present. Although the GST is a reliable alternative test for ITT, it should be cautiously used in the elderly because this population may have co-morbidities including vascular and cardiac diseases that could be potentiated with side effects of the test, such as severe hypotension [48]. Thus, several contraindications of ITT may also be considered as contraindications for GST in the elderly, such as seizure disorders and ischemic heart disease [48].

**Table 2:** Literature data on the effects of body mass index (BMI), age, and gender on the GH response to glucagon stimulation test (GST)

Reference	Subjects studied	BMI	Age	Gender
Gomez et al. Clin Endocrinol [40]	73 pts with pituitary disease and 46 NS	Yes	Yes	NA
Conceição et al. J Endocrinol Invest [41]	33 pts with pituitary disease and 25 NS	NA	No	No
Micmacher et al. Arq Bras Endocrinol Metab [55]	27 NS	No	NA	NA (study performed only in male gender)
Berg et al. Eur J Endocrinol [39]	49 pts with pituitary disease	No	No	NA
Toogood et al. Endocrine Pract [11]	952 pts enrolled in KIMS database	Yes	No	Yes
Yuen et al. Pituitary [33]	515 pts with pituitary disease	Yes	Yes (FD GST), No (WB GST)	NA
Dichtel et al. J Clin Endocrinol Metab [36]	20 pts with TPS, 20 pts with PPD and 47 NS	Yes	No	NA (study performed only in male gender)
Diri et al. Pituitary [37]	216 pts with pituitary disease and 26 NS	Yes	Yes	No
Simsek et al. Clin Endocrinol [62]	129 pts with pituitary disease	NA	NA	Yes
Hamrahian et al. Pituitary [42]	28 pts with pituitary disease and 14 NS	No	Yes (FD GST), No (WB GST)	No
Wilson et al. Growth Horm IGF Res [32]	42 pts with pituitary disease	Yes	No	No
Winter-Tavares et al. Endocrine [51]	42 old NS	No	No	NA
Casamitjana et al. J Endocrinol Invest [45]	34 PWS pts	Yes	No	NA
Akkar et al. Endocrine [28]	26 NS	Yes	NA	NA

*FD* fixed dose regimen; *GST* glucagon stimulation test; *KIMS* Pfizer International Metabolic Database; *NS* normal subjects; *NA* not available; *PPD* partial pituitary deficiency; pts patients; *PWS* Prader-Willi syndrome; *TPD* total pituitary deficiency; *WB* weight base regimen

Similarly to age, to date it is not yet clear if the GH response to the GST is sex dependent [61] (Table 2): analyzing 952 GSTs reported in the KIMS database, Toogood showed that women had significantly higher response than men [11]; in their study on 129 patients with pituitary disorder, Simsek et al. [62] reported that GH response to GST was significantly higher in female subjects; on the other hand, the peak GH response in women was not significantly higher than in men in the study by Wilson [32], but, overall women prescribed oral estrogen trended towards having a higher peak GH response than women not prescribed an oral estrogen [32], suggesting that the use of oral estrogens should be taken into consideration when interpreting results in females [32]. Anyway, to date, no study has specifically investigated the need to differentiate the cutoffs to GST as a function of gender.

There has been conflicting data in the literature as to whether there is a correlation between the peak GH response and the fasting baseline, peak, nadir, or rate of change in the BG following GST. Although it has been previously reported that with GST there is not a significant association with BG response [18], this has not been studied in detail in patients with higher than average BG nadir, and it is possible that these patients may not respond appropriately to GST.

## Future perspectives

In view of the limitations of the GST studies mentioned in the previous section, it would be appropriate to validate the GST exclusively in subjects with hypothalamic pituitary disease with (*cases*) and without (*controls*) GHD defined by a clinical point of view. Indeed, if the diagnostic threshold used for a particular test is inappropriately high (as it happens when comparing patients definitely affected by GHD with normal healthy subjects), then the patients it identifies may not have severe GHD and clinical consequences could be less severe. As a consequence, the long-term outcome of these patients may be different from those who do have severe GHD and these patients might perceive less benefit from GH replacement therapy; moreover, significant numbers of such patients could compromise the accuracy of outcome data derived from postmarketing surveillance studies [4].

It must be taken into account, that the proportion of patients with low GH response to provocative tests increases with the number of other pituitary hormone deficiencies and several studies involving panhypopituitary patients have shown that under certain circumstances GH stimulation tests may be unnecessary to diagnose GHD [3, 49–51]; therefore the remaining pituitary function can be used as a valid gold standard to establish the presence or absence of GHD. We have recently used this approach to reevaluate the BMI related cutoffs to GHRH + ARG test [4] demonstrating the need to review the present cutoffs [5] to reduce false positive diagnoses of GHD in adults.

Considering the conflicting data regarding a hypothetical relationship between age or gender and the GH response to GST, future studies will have to specifically evaluate the need to differentiate the diagnostic cutoffs according to these parameters. The evaluation and validation of specific diagnostic cutoffs to GST is particularly urgent in the transition phase and in the advanced age or in particular pathological conditions such as Prader–Willi syndrome to date too little studied [45].

Considering that a less robust hypoglycemic stimulus may contribute to an impaired GH response to GST as reported by Wilson et al. [32], it has been suggested measuring BG levels during GST to assist with clinical interpretation of GH dynamics, particularly in obese patients [32]; however, no certain nadir BG levels during GST has never been identified in order to guarantee the correct interpretability of the test. Furthermore, the diagnostic accuracy of the GST in patients with known disorders in glucose metabolism and those taking anti-diabetic agents deserves further study.

Finally, as already mentioned above, we recently reported significant hypokalemia in more than 50% of the tested subjects as an unexpected side effect to intranasal glucagon administration [34]. To date, there are no reports of hypokalemia induced by im or sc glucagon administration, but it is not clear whether this is due to a greater safety of these administration routes compared to intranasal one or simply because the trend of potassium levels during the execution of GST has never been evaluated.

## Conclusions

In the last few years, several studies have illustrated evolving trends in the use of GST over time as well as the need for increased education in GST use and interpretation throughout the endocrine community to standardize diagnostic and treatment practices according to current management guidelines [10–12]. Several studies have examined the accuracy and safety of the GST, and despite a lack of certainty about the mechanism by which glucagon stimulates GH release, the GST is the current de facto alternative to the ITT. However, it should be considered that a shortage of parenteral glucagon has recently been reported, at least in some countries (https://www.aifa.gov.it/documents/20142/1804899/Determina_DG-341-2023_Determina_blocco_esportazioni.pdf). If the production of parenteral glucagon ceases, as has already happened with the GHRH, it will be necessary urgently to seek further alternative tests to the ITT in the near future to correctly diagnose GHD in adults.

## Data Availability

A data availability statement is not applicable since this manuscript reviews current literature regarding the glucagon stimulation test in diagnosing adult GH deficiency.
